# Vitamin D and Vitamin D-Binding Protein in Health and Disease

**DOI:** 10.3390/ijms24054642

**Published:** 2023-02-28

**Authors:** Charlotte Delrue, Marijn M. Speeckaert

**Affiliations:** 1 Department of Nephrology, Ghent University Hospital, 9000 Ghent, Belgium; 2 Research Foundation-Flanders (FWO), 1000 Brussels, Belgium

Vitamin D is a fat-soluble secosteroid that exists in two forms: vitamin D_2_ and vitamin D_3_ [1]. While plants and fungi produce vitamin D_2_ from ergosterol [2], human skin produces vitamin D_3_ from 7-dehydrocholesterol (7-DHC) when exposed to UVB radiation from the sun [3]. The UV photons trigger a photochemical reaction that converts 7-DHC into previtamin D_3_, which then becomes vitamin D_3_ via a series of thermal isomerizations [4]. Vitamin D_3_ is transported to the liver and converted by CYP2R1 to 25-hydroxyvitamin D_3_ [25(OH)D_3_], the major circulating form of vitamin D_3_ [5,6]. Finally, 25(OH)D_3_ is converted to its active form, 1,25-dihydroxyvitamin D_3_ [1,25(OH)_2_D_3_], in the kidneys via CYP27B1 [7]. The primary function of 1,25(OH)_2_D_3_ is to maintain normal calcium levels and bone development by regulating phospho-calcium metabolism. Additionally, because vitamin D receptors (VDRs) have been discovered in numerous tissues (prostate, brain, breast, pancreas, colon, and immune cells), vitamin D can also provide non-skeletal actions. 1,25(OH)_2_D_3_ can play a role in the immune function and regulation of cell proliferation and differentiation in a variety of cell lineages (lymphocytes, endothelial cells, osteoblasts, and keratinocytes). Vitamin D can modify the risk of cardiometabolic outcomes, including hypertension, cardiovascular disease, and diabetes mellitus. Although the regulatory role of vitamin D in many biological processes is well documented, there is insufficient evidence to support the therapeutic use of vitamin D supplementation for the prevention or treatment of immunoinflammatory, infectious, or hyperproliferative diseases. 1,25(OH)_2_D_3_ exerts most of its functions via nuclear VDR, which forms a heterodimer with the retinoid X receptor (RXR). VDR/RXR in turn binds to vitamin D response elements (VDRE) in target genes and regulates gene transcription. The VDR pathway interacts with other intracellular signaling pathways to exert its biological effects (Figure 1). In some cases, 1,25(OH)_2_D_3_ exerts its influence without affecting gene expression or protein synthesis [8].

Vitamin D and its metabolites are generally transported from the site of production to target tissues. Mainly responsible for this transport is the vitamin-D-binding protein (DBP) or group-specific component (Gc-globulin), which binds 85% of the circulating 25(OH)D_3_ and 1,25(OH)_2_D_3_. It has a high binding affinity for vitamin D and its metabolites, which contributes to the maintenance of adequate vitamin D levels in the blood. On the other hand, albumin has a low binding affinity for vitamin D but also contributes to vitamin D transport, especially at low DBP levels. The relative proportion of vitamin D bound to DBP and albumin may affect the bioavailability of vitamin D, with vitamin D bound to DBP being more bioavailable than that bound to albumin. Understanding the role of these two carriers in vitamin D transport and regulation is critical for a better understanding of vitamin D metabolism and for developing effective strategies to treat vitamin D deficiency and related diseases. Aside from its sterol-binding capacity, DBP has been shown to possess antioxidant and anti-inflammatory properties, without binding to vitamin D, that likely contribute to its role in disease prevention and treatment. DBP has been found to regulate immune function and play a role in the removal of toxins and pathogens from the bloodstream. DBP is also involved in the regulation of calcium metabolism, which is critical for maintaining healthy bones, and may have anti-cancer properties in the form of the vitamin-D-binding protein macrophage-activating factor (DBP-MAF) (Figure 2) [9].

Seven interesting manuscripts (three original articles and four review articles) were included in the Special Issue titled “Vitamin D and Vitamin D-Binding Protein in Health and Disease”. In the first paper, Xu et al. [10] investigated how 1,25(OH)_2_D_3_ can affect the regenerative ability of intestinal stem cells. A novel approach was used to deliver regionally high concentrations of 1,25(OH)_2_D_3_ only to the inflamed intestine. Throughout the epithelial regeneration process, a pulse-and-chase method using 5-bromo-2′-deoxyuridine (BrdU) labeling was used to track Lgr5^+^ stem cells. Macrophages engineered to synthesize high levels of 1,25(OH)_2_D_3_ de novo specifically migrated into the inflamed intestine and supported the regenerative capacity of intestinal stem cells by hosting CD11b^+^Gr1^+^ macrophages. The results of this study may contribute to the development of a promising therapy to accelerate the repair of the intestinal epithelium in patients with inflammatory bowel disease (IBD). The second paper [11] addressed the free-hormone hypothesis for vitamin D in psoriasis patients. In a retrospective cross-sectional study involving 40 bionaive patients with mild-to-severe plaque psoriasis, total 25(OH)D_3_ serum levels appeared to be a reliable indicator of vitamin D status, as they correlated well with free 25(OH)D_3_ serum levels. No associations were found between any of the vitamin D metabolites and psoriatic disease severity. Despite potential confounding variables, such as age, sex, and body weight, DBP serum levels were higher in patients who self-reported having arthropathy than in those who did not. DBP may be a brand-new indicator of inflammation in psoriasis, particularly psoriatic arthritis.

As mentioned in the introduction, DBP performs a number of other roles in addition to binding to vitamin D, including therapeutic properties against tumors after conversion to DBP-MAF. Dolgova et al. [12] evaluated the benefit of the DBP-MAF-related factor (DBP-MAF-RF) in a Lewis carcinoma model under two clinical conditions: untreated tumor lesions and tumor resorption after “Karanahan” (derived from Sanskrit “killing the source”) therapy. The results of the study suggest that the primary requirement for the manifestation of the antitumor effect of DBP-MAF-RF is the inhibition of the suppressor effect of tumor-associated stroma (myeloid-derived suppressor cells, regulatory T lymphocytes). The anti-inflammatory effect of DBP-MAF-RF supports tumor progression and immune surveillance defense when the tumor has an active protective humoral background. The pro-inflammatory tumor reactive effects of DBP-MAF-RF become more pronounced when the tumor loses the suppressive humoral background. The results of this study demonstrate that DBP-MAF-RF, when used properly in conjunction with the “Karanahan” strategy, may enhance the antitumor immune response elicited by “Karanahan” therapy.

As discussed in two reviews [13,14] in this Special Issue, diabetes mellitus, renal dysfunction, and heart failure act synergistically. Diabetes mellitus and its complications have been associated with vitamin D deficiency, although the major question remains whether vitamin D deficiency is a cause or merely a consequence of diabetic kidney disease (DKD). The possible renal protective role of vitamin D in the development of DKD and the reversal of pre-existing renal damage is not known, although vitamin D has been shown to play a critical role in the development of DKD in numerous animal and human studies. More extensive, protracted, randomized, controlled human studies are needed to determine how vitamin D and its analogues may affect mortality, end-stage kidney disease (ESKD) progression, the development of DKD, and the decline in estimated glomerular filtration rate (eGFR) [14].

In another article, Chan et al. [15] provided a systematic review of the association between vitamin D and various ocular diseases. In addition to the association with diabetic retinopathy, vitamin D was also associated with myopia, age-related macular degeneration, and dry eye syndrome. Evidence for an association with other ocular diseases such as glaucoma, thyroid disease, and retinoblastoma is still sparse. Although vitamin D has been associated with several disease-related processes, the causes of these diseases are complex, and knowledge of their underlying mechanisms remains limited. Vitamin D not only has an impact on the homeostasis of mineral metabolism, but also has anti-inflammatory and antioxidant properties. It influences the process of anti-angiogenesis by modulating various aspects of the cell cycle, such as cell division, proliferation, and apoptosis. Ocular tissues contain the vitamin D receptor (VDR) and vitamin-D-regulating enzymes, and research has shown that these tissues can activate and regulate vitamin D, indicating the importance of vitamin D in maintaining eye health.

Finally, Papagni et al. [16] studied the antifungal effect of vitamin D. Due to its relationship with oxidative balance, vitamin D inhibits the replication of Mycobacterium tuberculosis in vitro and has shown promise for the treatment of tuberculosis. Further clinical studies are needed to evaluate the efficacy of the drug in vivo, rule out confounding factors, and ultimately determine the dosages and delivery systems best suited for the treatment and prophylaxis of tuberculosis. It is important to investigate the potential benefits of combining vitamin D with other vitamins, such as vitamin A, which has antifungal activity in vitro. On the other hand, it would be advisable to measure vitamin D levels in both patients with active tuberculosis and those at risk, and to administer vitamin D, at least to achieve adequate levels, based on evidence of its efficacy in vitro and taking into account its low incidence of side effects, even at high doses, and its low cost.

In summary, this Special Issue on vitamin D and its binding proteins showcases a promising role in monitoring, preventing, and treating a variety of human diseases, including IBD, psoriasis, tuberculosis, diabetic nephropathy and retinopathy, and cancer. However, more research is needed to fully understand the mechanisms by which vitamin D affects these diseases and to determine the optimal vitamin D levels needed to prevent and treat disease. Future studies will also explore the potential use of DBP as a biomarker for disease and as a therapeutic target. The discovery of the unique role of vitamin D and its binding protein in human health has the potential to lead to new and innovative treatments for a variety of diseases, making it an exciting and rapidly evolving area of research.

## Figures and Tables

**Figure 1:**
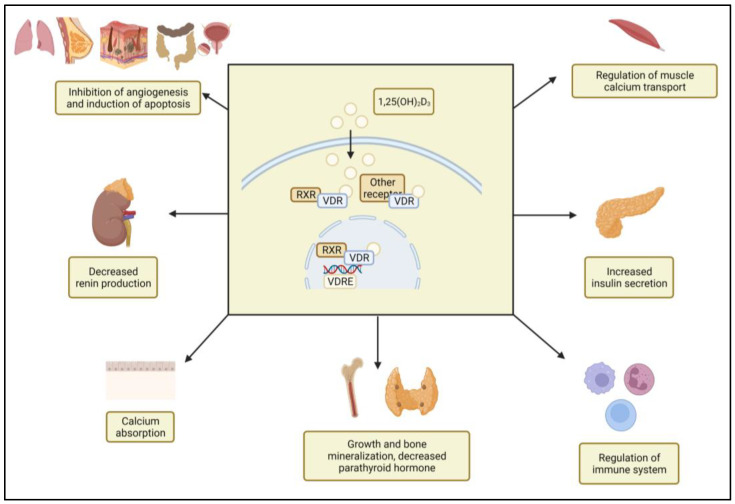
Vitamin D exerts its biological effects through the vitamin D receptor (VDR). 1,25(OH)_2_D_3_ can quickly diffuse through cell membranes and bind to VDR. VDR forms heterodimers with the retinoid X receptor (RXR) after binding to the ligand, moving into the nucleus where it binds to vitamin D response elements (VDREs) to regulate gene transcription. The VDR also controls the transcription of genes by interacting with other nuclear receptors. The functions of vitamin D include growth and bone mineralization, immune function regulation, insulin secretion regulation, cell proliferation control, cell differentiation stimulation, apoptosis induction, phosphor–calcium homeostasis regulation, and muscle calcium transport regulation.

**Figure 2:**
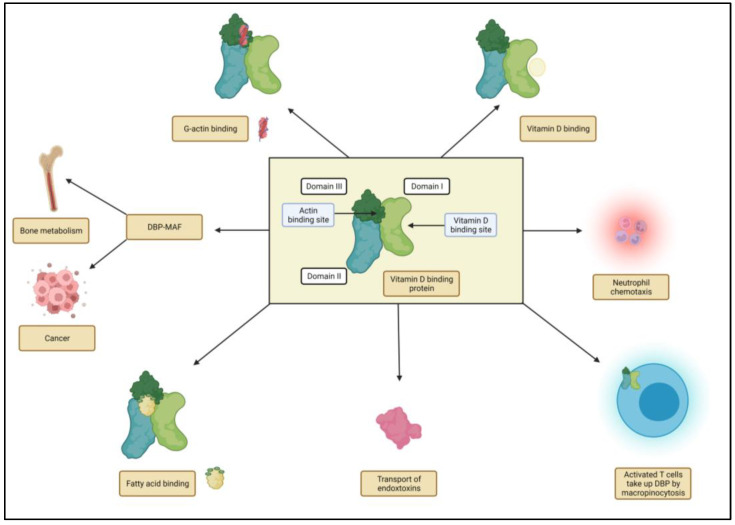
An overview of vitamin-D-binding protein’s various physiological roles, including the binding of vitamin D metabolites, the binding of fatty acids, the transport of endotoxins, neutrophil chemotaxis, the influence on T cell response, actin scavenging, and the influence of vitamin-D-binding protein-macrophage activating factor (DBP-MAF) on bone metabolism and cancer.

## Data Availability

Not applicable.
